# Exploring Machine Learning for the Curation of DNA Barcode Reference Libraries: A Case Study With Marine Fish Species of the Eastern Atlantic and Adjacent Waters

**DOI:** 10.1111/1755-0998.70223

**Published:** 2026-11-08

**Authors:** João T. Fontes, André O. Ferreira, Ana S. Lavrador, Pedro Soares, Filipe O. Costa, Carlos A. Canchaya

**Affiliations:** ^1^ Centre of Molecular and Environmental Biology (CBMA) / ARNET‐Aquatic Research Network, Department of Biology University of Minho Braga Portugal; ^2^ Institute of Science and Innovation for Bio‐Sustainability (IB‐S) University of Minho Braga Portugal; ^3^ Department of Biochemistry, Genetics, and Immunology University of Vigo Vigo Spain; ^4^ Marine Research Centre (CIM‐UVIGO) Universidade de Vigo Vigo Spain

**Keywords:** automation, curation, DNA barcoding, DNA metabarcoding, eDNA, machine learning, marine fish, species‐level identification

## Abstract

Accurate species assignments using (e)DNA (meta)barcoding depend on the reliability of DNA barcode reference libraries. Manual curation can help resolve pseudo‐discordances and identify incongruences, but it is time‐consuming and increasingly difficult to scale as reference sequence data continue to expand. We explored the potential of automated approaches to support the curation of DNA barcode records within discordant Barcode Index Numbers (BINs). Records were mined from the Barcode of Life Data System (BOLD) for marine fish species occurring in the Eastern Atlantic and adjacent waters, and features describing taxonomic consistency, morphospecies‐BIN congruence, and associated metadata were extracted. Within a subset comprising 52,207 records, 3055 species names, and 1138 discordant BINs, each record was manually classified as *supported* or *inconclusive* using the taxonomic and metadata information associated with the records as a heuristic guide. We compared a simple rule‐based approach with logistic regression, extreme gradient boosting (XGBoost) and artificial neural networks (ANNs). The rule‐based approach achieved accuracies ranging from 80.4% to 90.9% across the evaluated thresholds. Logistic regression achieved the highest overall accuracy (92.1%), followed closely by ANN (91.7%) and XGBoost (91.4%). These results support the use of machine‐learning approaches to assist the triage of discordant records by helping identify cases that warrant closer human inspection. Such approaches could be integrated into a human‐in‐the‐loop framework that retains manual assessment where taxonomic judgement is required, while helping to scale the screening and curation of rapidly growing DNA barcode reference libraries.

## 1 Introduction

Reference sequence libraries are the basis for taxonomic identification through (e)DNA (meta)barcoding approaches. The accuracy of reference sequence data is necessary for incorporating molecular identification tools into biodiversity monitoring, conservation and fisheries management. Yet, misidentifications and ambiguities commonly emerge within public databases and can compromise the accuracy of taxonomic assignments (Cheng et al. 2023; Keck et al. 2023; Packer et al. 2009; Pentinsaari et al. 2020). This is especially pertinent for (e)DNA metabarcoding studies, where large numbers of sequence reads are automatically queried against reference sequence data (Bengtsson‐Palme et al. 2018; Kirse et al. 2021; Lavrador et al. 2023). Errors and ambiguities in reference libraries may arise at different stages of the sequence generation workflow, including (a) the generation of incomplete or low‐quality sequences from degraded DNA (e.g., old museum specimens), (b) sequencing artefacts (e.g., incorrect base calls or indels), and (c) co‐amplification of non‐target DNA, including microbial or environmentally associated DNA and NUMTs (nuclear DNA sequences of mitochondrial origin), all of which can introduce noise into the data and complicate downstream taxonomic interpretation (Collins et al. 2019; Hebert et al. 2023; Marsh et al. 2025; Nugent et al. 2020). Nonetheless, the assignment of an incorrect species name to a sequence—through morphological misidentification, mislabelling or cross‐contamination—remains a major source of incongruence (Grant et al. 2021; Keck et al. 2023). Several approaches have been developed to reduce the impact of such errors and inconsistencies (Antil et al. 2023; Claver et al. 2023; Ershova et al. 2023; Gostel and Kress 2022), but preventing taxonomic misidentifications from entering public reference libraries remains challenging.

Existing tools address different aspects of reference‐library compilation and curation. Software such as *crabs* (Jeunen et al. 2023), *rCRUX* (Curd et al. 2024), and *refdb* (Keck and Altermatt 2023) implement robust data mining and quality control procedures, including the detection of incomplete or inconsistent taxonomic annotations. Packages such as *coil* (Nugent et al. 2020) and *debar* (Nugent et al. 2021) focus instead on the reliability of cytochrome c oxidase I (COI) sequences, applying profile hidden Markov models (PHMMs) to detect indels and related artefacts. Other systems aim to assemble comprehensive libraries: mkCOInr (Meglécz 2023) dereplicates barcode data from the Barcode of Life Data system (BOLD; Ratnasingham and Hebert 2007) and NCBI GenBank (Sayers et al. 2025) while controlling for synonyms; while Meta‐Fish‐Lib (Collins et al. 2021) integrates multiple markers and includes steps to visually flag potentially misidentified records. More recently, BOLDistilled (Prosser et al. 2025) introduced an automated approach for constructing compact, versioned COI reference libraries from BOLD by reducing sequence redundancy within Barcode Index Numbers (BINs). Taken together, these tools provide complementary solutions, but the identification and resolution of taxonomic ambiguities remain difficult to scale as reference databases continue to grow.

The BIN System (Ratnasingham and Hebert 2013) implemented in BOLD, is central to ensuring the reliability of DNA barcode reference libraries. This system clusters COI barcode sequences in a manner that, ideally, mirrors the delimitation of morphospecies. It provides an operational framework for grouping genetically similar sequences and comparing molecular clusters with species‐level taxonomic assignments. BINs partition sequences into mutually exclusive units that can result in different outcomes (Ratnasingham and Hebert 2013): MATCH, when a BIN corresponds to a single morphospecies; MERGE, when two or more morphospecies are assigned to the same BIN (i.e., a discordant BIN, which may reflect limited resolution of the COI marker or misidentifications); SPLIT, when records assigned to a single species are divided across multiple BINs—sometimes indicating cryptic diversity or population structure; and MIXTURE, when sequences from a particular species are found in both concordant and discordant BINs (often as a result of errors). These four categories provide a solid basis for detecting taxonomic inconsistencies and promoting appropriate curation practices. However, the BIN system is highly dynamic: the addition of new records can cause BINs to merge or split. Moreover, the algorithm that underpins this flexibility is not publicly available, which can limit the interpretability of BINs in some applied contexts, such as forensic or regulatory work. In these scenarios, static and voucher‐linked reference libraries are often preferred (e.g., the Reference Standard Sequence Library for Seafood Identification, RSSL; FDA 2024).

The occurrence of species names assigned to discordant BINs (i.e., MERGE or MIXTURE scenarios) is frequent for several taxonomic groups both due to operational and biological reasons (Fontes et al. 2021; Lavrador et al. 2023; Oliveira et al. 2016; Radulovici et al. 2021). Although BOLD offers users a tool to highlight and further examine these BINs given a set of available records, their curation remains unresolved. Costa et al. (2012) developed and tested a morphospecies‐centred qualitative grade system to assess the congruency between morphospecies and sequence clusters, using marine fishes from Portugal as a case study. Oliveira et al. (2016) updated this system to use BINs as molecular operational taxonomic units (MOTUs) and audited a reference library for European marine fish species using this approach. Later, Fontes et al. (2021) automated the process by developing BAGS (Barcode, Audit & Grade System), an R‐based framework developed to mine public records from BOLD and assign grades according to the congruence between species names and BINs at a given point in time. Although this approach can highlight concordant correspondences between morphospecies and BINs, as well as species names that are assigned to at least one discordant BIN, it does not differentiate between pseudo‐discordances (e.g., two synonym species names sharing a BIN) and actual inconsistencies, such as potential misidentifications. In addition, it does not examine discordant BINs to assess the plausibility of species assignments. More recently, the Biodiversity Genomics Europe (BGE) Library Curation Tool (Kühbandner et al. 2026) has provided a dedicated environment for the expert curation of DNA barcode records, enabling taxonomic experts to review and annotate individual records within discordant BINs, revise taxonomic information, and document curation decisions.

The curation of discordant BINs requires accounting for pseudo‐discordances caused by ambiguous names or synonymy, as well as cases arising through operational errors or limited molecular discrimination among closely related morphospecies. Such curation is particularly important when the research aims for species‐level identification, where the ideal scenario is for each BIN to correspond to a single species name. In practice, achieving this level of data curation requires researchers to make informed judgement calls, inspecting records' data and metadata to segregate those deemed well‐supported from those considered ambiguous (Ferreira et al. 2024; Lavrador et al. 2023; Magoga et al. 2022; Nilsson et al. 2018; Radulovici et al. 2021). While such a curation approach does not guarantee absolute accuracy, it is often essential for preventing discordant records from undermining downstream analyses. This case‐by‐case approach is notoriously time‐consuming, and the challenge scales considerably with dataset size. Moreover, because DNA barcodes are continually added to BOLD, previously curated BINs may require re‐evaluation as new inconsistencies emerge. These challenges are particularly critical for (e)DNA metabarcoding, where high‐throughput sequencing (HTS) produces vast numbers of reads that rely on accurate assignment to reference sequences for accurate species identification. Workflows like the Multiplex Barcode Research And Visualization Environment (mBRAVE; Ratnasingham 2019), which can assign HTS reads to BINs using BOLD reference data, emphasize the importance of reliable reference datasets for metabarcoding.

Given the millions of reference sequences already publicly available and the continued rapid growth of reference databases, automated approaches may help address these challenges. On one hand, rule‐based approaches provide a straightforward and effective way of structuring curation decisions (Lavrador et al. 2023). However, fixed rules may not capture all combinations of evidence considered during case‐by‐case assessment. Machine learning models offer a complementary approach by learning from previously curated examples and approximating how this information is combined in manual decisions (Jordan and Mitchell 2015). Their performance nevertheless depends on the quality and representativeness of the training data, and some approaches can be difficult to interpret (Mehrabi et al. 2021; Rudin 2019). Rather than replacing manual curation, such models may therefore be most useful for supporting triage and prioritizing records for human review.

In this study, we evaluated different levels of automation for manual BIN curation, beginning with a simple rule‐based approach and logistic regression as baseline classifiers, and extending the comparison to artificial neural networks (ANNs) and extreme gradient boosting (XGBoost). Logistic regression models the probability of a binary outcome as a function of a linear combination of predictor variables, providing a simple and readily interpretable classification framework (Cox 1958). Artificial neural networks (ANNs), in contrast, consist of layers of interconnected nodes that can learn complex nonlinear relationships from data (Goodfellow et al. 2016), while XGBoost is a tree‐based ensemble method that builds multiple decision trees in sequence, each one correcting the errors of the previous to achieve strong predictive performance (Chen and Guestrin 2016). This comparison allowed us to test whether more complex models matched manual curation more closely than simpler approaches.

For this purpose, we used a DNA barcode reference library comprising marine fish species occurring in the Eastern Atlantic Ocean and adjacent waters. Marine fishes have been increasingly targeted in (e)DNA (meta)barcoding studies (Xiong et al. 2022; Ye et al. 2025) and comprise a group that is relatively prone to taxonomic ambiguities and BIN discordances (Claver et al. 2023; Fontes et al. 2021; Oliveira et al. 2016). Being well represented in BOLD, marine fishes provided a dataset large enough to train and evaluate the different approaches, yet manageable for case‐by‐case data curation. Our main objective was to assess how closely automated approaches could approximate manual classification of discordant DNA barcode records, and to evaluate their potential to scale and support reference library curation.

## 2 Methodology

An overview of the workflow utilized is shown in Figure 1.

**FIGURE 1:**
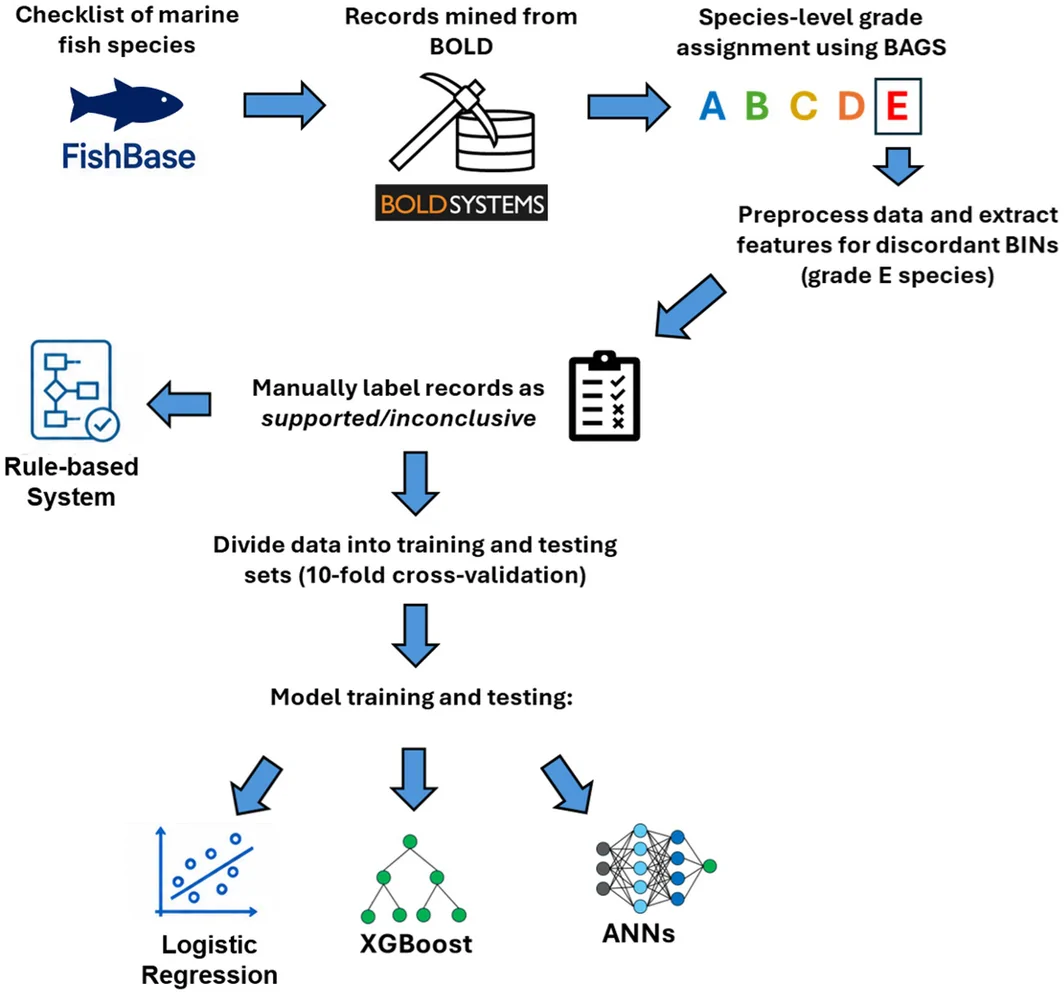
Overview of the workflow utilized for testing the use of different automated methods in the curation of discordant BINs.

### 2.1 Data Mining

We assembled a list of fish species occurring in the Eastern Atlantic Ocean and adjacent waters (e.g., Greenland Sea, Mediterranean Sea) from FishBase (Froese and Pauly 2023), resorting to the *rfishbase* R package version 4.1.2 (Boettiger et al. 2012) on December 21, 2023, using both ecoregion and country‐level queries (Table S1). Subsequently, we removed species annotated with ‘error’, ‘misidentification’ or ‘questionable’. We then accessed the World Register of Marine Species (WoRMS Editorial Board 2023) through the *worms* R package version 0.2.2 (Holstein 2018) on December 21, 2023, to standardize the taxonomic information and retain only marine species according to the database's habitat assignments. Species occurring in both marine and brackish/freshwater environments were included. Using the assembled species list as a query, we adapted the code from BAGS to mine and annotate the respective DNA barcode records from BOLD version 4 API (Ratnasingham et al. 2024) on January 18, 2024. Contrary to the standard BAGS pipeline, our database contained all records regardless of their sequence quality, except for records without species‐level annotation or assigned BINs. In addition, we expanded our data mining to include all records with species‐level annotation associated with BINs linked to the species in our checklist (regardless of the geographical distribution of these additional species). This step ensured complete representation of each BIN, which served as the units of taxonomic congruence in this study.

### 2.2 Data Preprocessing and Feature Extraction

Most of the data preprocessing and feature extraction steps were conducted using the R programming language (R Core Team 2024) version 4.1.2, within the integrated environment RStudio (Allaire 2012) version 2021.9.1.372. Qualitative grades, from A to E, were assigned to each species in the dataset according to the congruence between species names and sequences clustered in BINs, as described by Fontes et al. (2021) and Oliveira et al. (2016):

- Grades A and B—species names assigned to a single BIN integrating solely records with that species name.
- Grade C—species names assigned to more than one BIN, albeit integrating only records from said species.
- Grade D—non‐discordant species names (i.e., non‐Grade E) with fewer than three records in the data set.
- Grade E—species names assigned to at least one BIN comprising more than one species name (i.e., the species name may occur in several BINs, but at least one of these BINs contains additional species names).

The dataset generated following grade assignment is available in Table S2. Considering that our goal was to segregate records based on their putative taxonomic reliability, the subsequent analyses focused exclusively on records from discordant BINs within Grade E species.

For each record in the discordant BINs subset associated with a specific species name clustered in a BIN, we extracted numerical and binary features. Numerical features included: the number of distinct species names within the BIN, the number of distinct BINs assigned to the species name, the total number of records for the species names in the dataset, the total number of records in the respective BIN, the number of records for that particular species name in the BIN, the percentage of records within the BIN represented by that species name, and the proportion of records belonging to the same genus in each BIN. We also calculated the Shannon entropy for each BIN to quantify the evenness of species name representation within a cluster (Pielou 1966). Moreover, we included the number of different individuals who identified at least one specimen of the species clustered in the BIN, as well as the number of distinct institutions housing vouchers of the species recorded in the BIN. For species names with no information on taxonomic identifier or housing institution in a particular BIN, the value was zero.

To account for pseudo‐discordances arising from synonymy or naming inconsistencies, we also determined two binary features: the first informed whether the species shared the BIN with at least one synonym species name, which was achieved through the WoRMS database resorting to the *taxize* R package version 0.9.99 (Chamberlain and Szöcs 2013); and the second checked whether the species name contains ambiguous expressions (e.g., sp., cf., complex., group., etc.) or does not follow the binomial nomenclature.

### 2.3 Manual Dataset Labelling

During the labelling process, we utilized the records' information mainly as a heuristic guide rather than establishing thresholds through a strictly rule‐based system. Each record was categorized as *supported* when the accumulated evidence and context from the features suggested that the record was correctly identified. In contrast, a record was labelled as *inconclusive* when potential misidentifications were detected, when multiple consolidated morphospecies were grouped in one BIN, or if the record's reliability remained inconclusive.

One heuristic criterion we considered was the predominance of a species assignment within a BIN (Ratnasingham and Hebert 2013; Prosser et al. 2025), together with other information such as the number of different identifiers and institutions associated with the records, the number of BINs assigned to each species name, and the number and identity of the species names occurring in the same BIN. For instance, if a BIN contained 95 records for species A, with the taxonomic identifications deriving from 10 individuals and specimens deposited across several institutions; and five records for species B, identified by only one individual and represented by material from a single institution, then the records for species A would be labelled as *supported*, while species B records would be labelled as *inconclusive*. In this case, the predominance of species A would be reinforced by broader support across both identifiers and voucher‐holding institutions. On the other hand, if a BIN featured 55 records from species A and 45 records from species B, where species A had been identified by 10 individuals and species B by eight, with both species represented across multiple institutions, all records would be considered *inconclusive*. Although species A has a greater number of records and identifiers, the difference is marginal, and neither the distribution of identifiers nor that of voucher material provides sufficiently contrasting support for one assignment over the other. Accordingly, all records would be classified as *inconclusive* as a conservative decision. Conversely, if in this scenario species A and species B were synonyms, all records would be labelled as *supported*. This is because the two species names are synonyms referring to the same taxon, meaning that the apparent discordance within the BIN results from nomenclatural differences rather than conflicting species assignments. The available features were considered collectively when assigning records as supported or inconclusive. The only rule‐based criterion we applied was classifying all records with ambiguous species names as *inconclusive* since they do not provide unambiguous species‐level identification. The labelled data set is available in Table S3.

## 3 Automated Classification Approaches

We evaluated four automated classification approaches: a simple rule‐based method, logistic regression, artificial neural networks (ANNs), and extreme gradient boosting (XGBoost). All analyses were implemented in Python version 3.11.5 using Spyder version 5.4.3 (Raybaut 2009). Logistic regression was implemented using scikit‐learn version 1.4.2 (Pedregosa et al. 2011), ANNs were built using TensorFlow version 2.10.0 (Abadi et al. 2016) through the Keras high‐level API (Chollet 2015), and XGBoost was implemented using the *xgboost* package version 2.0.3 (Chen and Guestrin 2016).

For the rule‐based approach, records associated with ambiguous species names were classified as *inconclusive*, whereas records in BINs whose discordance resulted exclusively from synonymous species names were classified as *supported*. For the remaining records, classification was based on the proportion of records assigned to each species name within the BIN: species assignments reaching a predefined majority threshold were classified as *supported*, while the remaining records were classified as *inconclusive*. Thresholds ranging from 55% to 95%, in 5% increments, were evaluated to examine the performance of increasingly stringent majority criteria.

For logistic regression, ANN, and XGBoost, model evaluation used 10‐fold grouped cross‐validation, with all records from the same BIN kept within the same fold and identical outer test partitions used across models. Within each outer training set, 20% of the BINs were reserved for internal validation. Candidate hyperparameter configurations were evaluated on this subset, and the configuration achieving the highest macro‐F1 was selected for each fold. The selected configuration was then retrained on the complete outer training set and evaluated on the held‐out test fold. Final performance was calculated from the combined out‐of‐fold predictions across the 10 test folds. All preprocessing transformations were fitted using only the training data and subsequently applied to the corresponding validation or test data. Percentage‐based predictors and Shannon entropy were standardized, while count‐based predictors were log1p‐transformed and, for logistic regression and ANN, subsequently standardized. Binary predictors were retained in their original form.

Hyperparameter tuning was performed independently for the three models. The complete search spaces are summarized in Table 1, while grid‐search results, fold‐specific selected configurations, and training and validation curves for ANN and XGBoost are provided in Data S1. ANN models used a sigmoid activation function in the output layer and were trained using the Adam optimizer and binary cross‐entropy loss. XGBoost models used a binary logistic objective, with logloss monitored during training. For all three supervised approaches, predicted probabilities greater than 0.5 were classified as *supported*, whereas values less than or equal to 0.5 were classified as *inconclusive*. Predictive performance was assessed using accuracy, precision, recall, macro‐ and weighted F1‐score, class‐specific metrics, and confusion matrices. Data S2 contains the record‐level predictions and additional model outputs, including fold‐specific results, test‐BIN assignments, fitted models and XGBoost permutation importance results.

**TABLE 1:** Hyperparameter search spaces evaluated for logistic regression, artificial neural network (ANN), and extreme gradient boosting (XGBoost) models.

Model	Hyperparameter	Values evaluated
Logistic regression	Regularization strength (*C*)	0.001, 0.01, 0.1, 1, 10, 100
	Solver	lbfgs, liblinear
	Class weighting	None, balanced
	Penalty	L2
	Maximum iterations	1000
ANN	Hidden layers	[16], [32], [64], [32, 16], [64, 32]
	Activation function	ReLU, ELU
	Learning rate	0.0001, 0.001, 0.003
	Batch size	16, 32, 64
	Epochs	10, 20, 30
XGBoost	Number of boosting rounds	75, 100, 125, 150
	Maximum tree depth	3, 4, 5
	Learning rate	0.01, 0.05, 0.1
	Subsample ratio	0.8, 1.0
	Column sampling	0.8, 1.0
	Minimum child weight	1, 5

The R and Python scripts used for data mining, preprocessing, feature extraction and model implementation are available at https://github.com/tadeu95/ML‐Curation‐DNA‐Barcodes.

## 4 Results

The mined dataset for marine fishes of the Eastern Atlantic and adjacent waters contained 66,432 records, representing 1995 species names and 3072 BINs. Expanding to species‐level annotated records belonging to the BINs in the initial set of records yielded a complete dataset (Table S2) of 83,473 records, comprising 3991 unique species names and 2956 accepted species names (i.e., after removal of previously described ambiguous names). The distribution of qualitative grades assigned to these species by BAGS is shown in Figure 2. Among these, 52,207 records corresponded to discordant BINs. Following manual curation, 27,034 records were labelled as *inconclusive*, and 25,173 records were labelled as *supported* (Table S3). The performance of the rule‐based approach varied considerably with the majority threshold applied (Table 2). Accuracy ranged from 80.4% at the 95% threshold to 90.9% at the 80% threshold, while macro‐F1 ranged from 79.4% to 90.9%. The highest observed performance was obtained at the 80% threshold, with both accuracy and macro‐F1 reaching approximately 90.9%.

**FIGURE 2:**
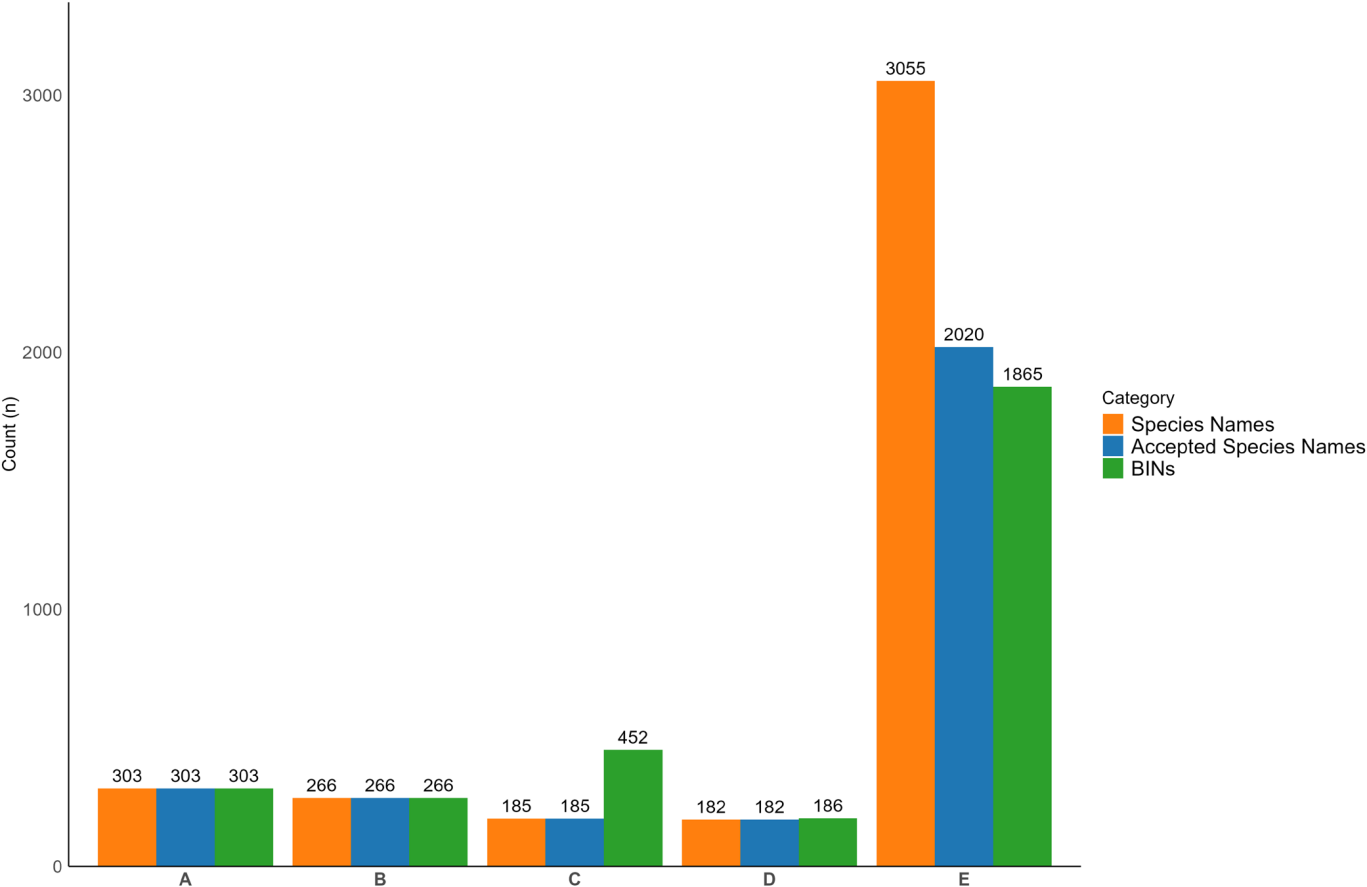
Distribution of the number of species names, accepted species names, and BINs in the dataset assigned through the BAGS auditing system to each qualitative grade (A: consolidated concordance; B: basal concordance; C: multiple BINs; D: insufficient data; E: discordant species assignment).

**TABLE 2:** Performance of the rule‐based classification approach across the evaluated majority thresholds.

Majority threshold (%)	Accuracy (%)	Macro‐F1 (%)
55	82.0	81.7
60	84.7	84.5
65	88.0	88.0
70	88.6	88.5
75	90.5	90.5
80	90.9	90.9
85	90.2	90.2
90	87.3	87.1
95	80.4	79.4

Among the supervised approaches, logistic regression showed the highest numerical overall out‐of‐fold performance, with both accuracy and macro‐F1 reaching approximately 92.1% (Table 3). ANN achieved approximately 91.7% for both metrics, followed by XGBoost at approximately 91.4%. Across the 10 held‐out outer folds, accuracy ranged from 83.3% to 98.4% for logistic regression (SD = 4.2%), 83.9% to 96.1% for XGBoost (SD = 4.3%), and 84.7% to 95.8% for ANN (SD = 4.3%; Figure 3). Class‐specific precision, recall, and F1‐scores are reported in Table 3, while confusion matrices for the three supervised approaches are shown in Figure 4. Permutation importance analysis of the XGBoost models showed that the proportion of records within a BIN assigned to the focal species was the most influential predictor, with a mean decrease in macro‐F1 of 0.323 when permuted across the 10 outer folds (SD = 0.047; Figure 5). The number of unique identifiers, synonym status, and Shannon entropy showed substantially smaller mean permutation importances, while the remaining predictors had comparatively limited individual effects.

**TABLE 3:** Performance of logistic regression, artificial neural network (ANN), and XGBoost based on the combined held‐out predictions from the 10‐fold grouped cross‐validation.

Metric	Class	Logistic regression	ANN	XGBoost
Accuracy	Overall	92.1	91.7	91.4
Macro‐F1	Overall	92.1	91.7	91.4
Precision	Inconclusive	94.5	92.3	91.2
	Supported	89.8	91.1	91.6
Recall	Inconclusive	90.0	91.6	92.3
	Supported	94.4	91.8	90.5
F1‐score	Inconclusive	92.2	92.0	91.7
	Supported	92.0	91.4	91.0

**FIGURE 3:**
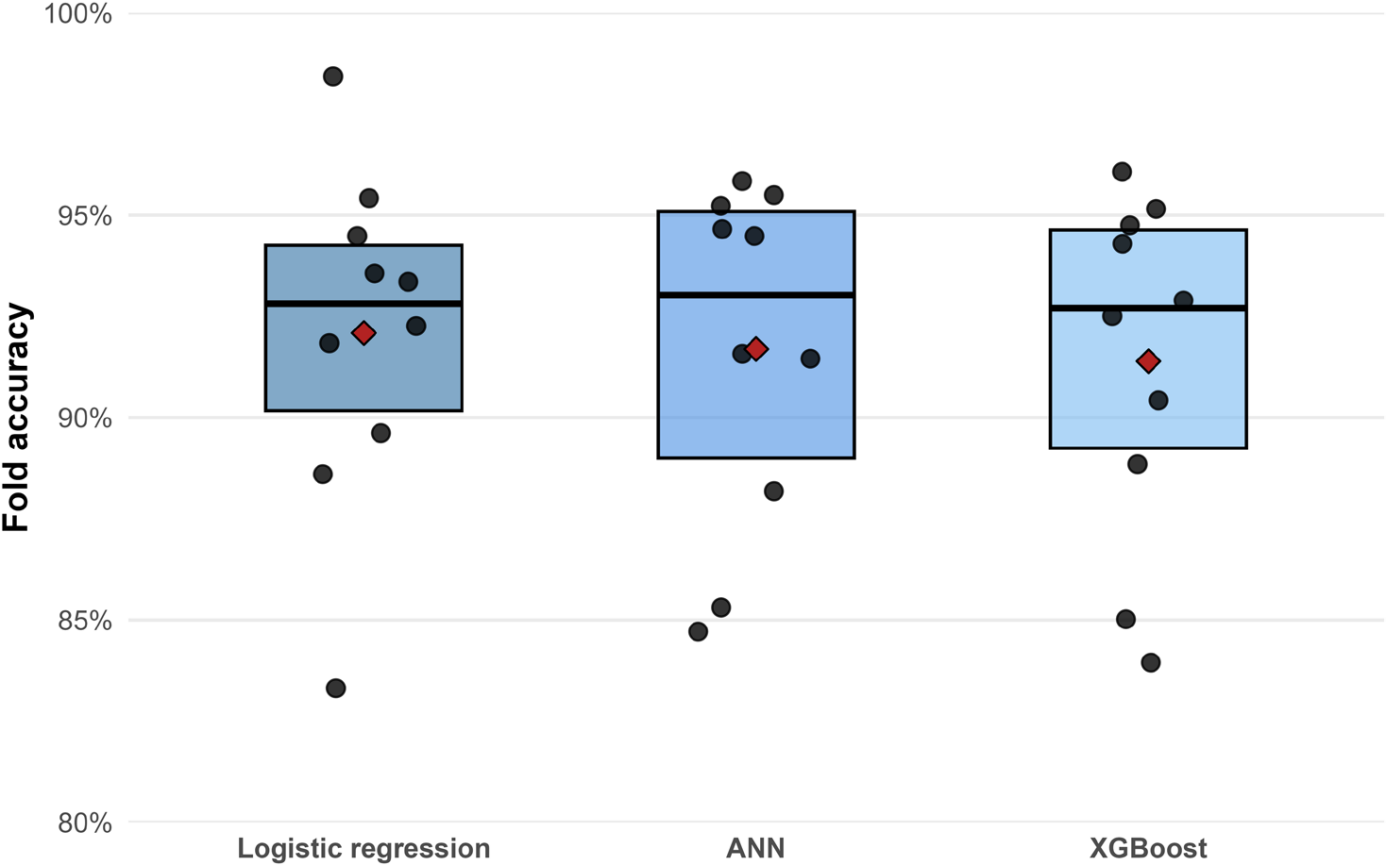
Cross‐validation accuracies across the 10 held‐out outer folds for logistic regression, artificial neural network (ANN), and XGBoost classifiers. Each point represents the accuracy obtained in one fold. Boxes indicate the interquartile range (IQR), horizontal lines indicate the medians, and red diamonds indicate the mean accuracy for each model.

**FIGURE 4:**
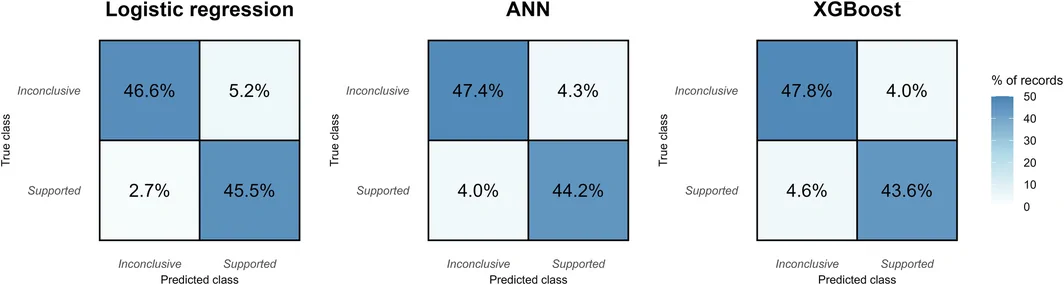
Confusion matrices for logistic regression, artificial neural network (ANN), and XGBoost based on the combined out‐of‐fold predictions from the 10‐fold grouped cross‐validation. Rows represent the manually assigned classes and columns the predicted classes.

**FIGURE 5:**
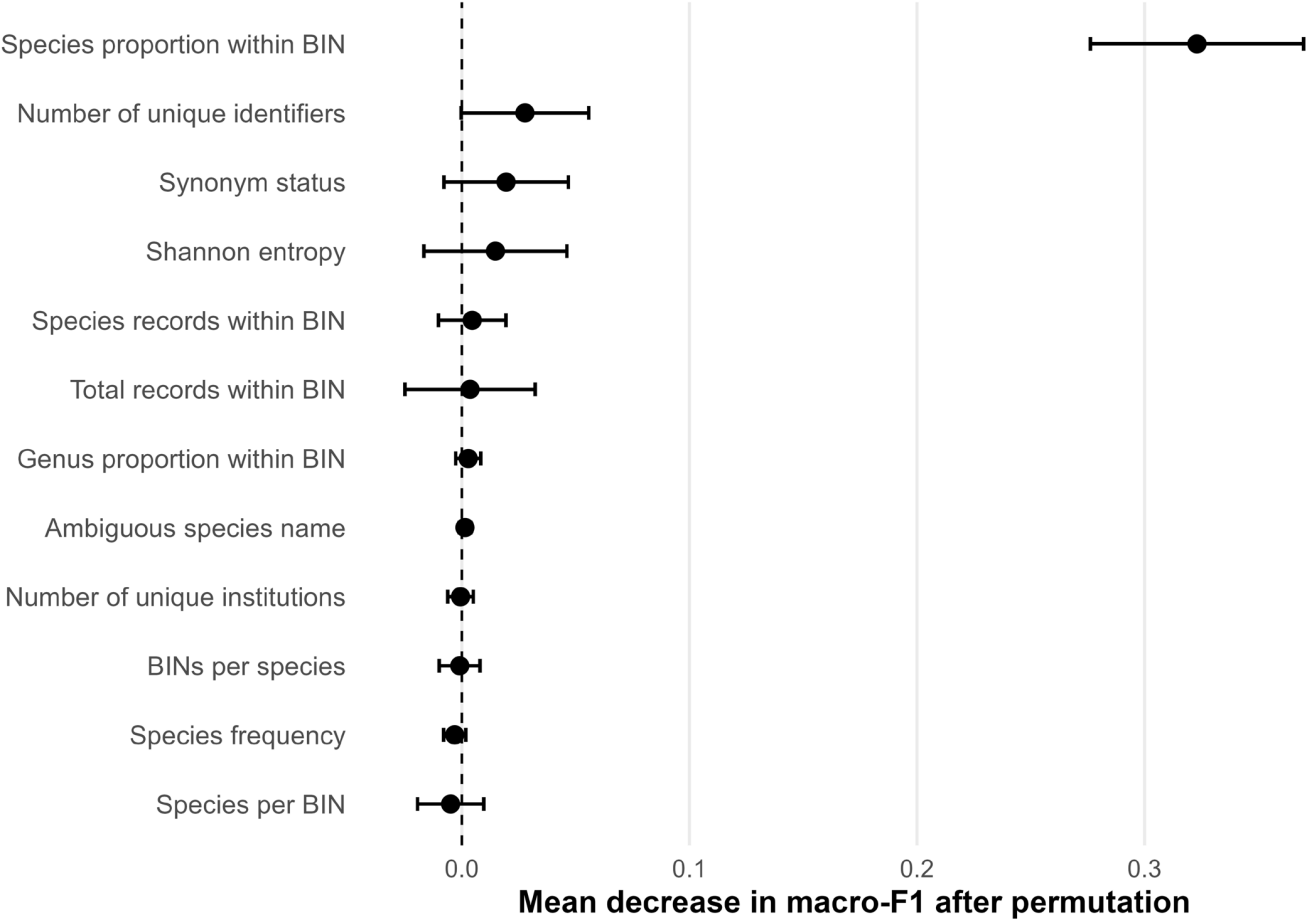
Permutation importance of predictors in the XGBoost models. Points represent the mean decrease in macro‐F1 following permutation of each predictor across the 10 held‐out outer folds, and error bars represent the standard deviation among folds. Larger positive values indicate a greater reduction in predictive performance when the corresponding predictor is permuted. The dashed line indicates zero permutation importance.

## 5 Discussion

The continuous expansion of DNA barcode reference libraries enhances their utility but also increases the challenge of maintaining taxonomic consistency, with errors and incongruences affecting the accuracy of taxonomic assignments (Collins et al. 2021; Keck et al. 2023). Currently, there are over 27 million barcodes in BOLD (accessed on 23 August 2026). As the database grows and new barcodes are assigned to BINs, managing these data becomes increasingly complex and inconsistencies are likely to accumulate over time. This is particularly relevant for fishes, for which species‐level identification is often a primary goal in (e)DNA (meta)barcoding studies (Cicala et al. 2024; Duhamet et al. 2023; Ferreira et al. 2024). Thus, using BINs as operational proxies for morphospecies requires a sufficiently clear correspondence between molecular clusters and species‐level taxonomic assignments.

The mined COI dataset for Eastern Atlantic fishes comprised a high proportion of species names assigned to at least one discordant BIN—Grade E species (Figure 2). Importantly, Grade E assignments should be interpreted as flags for closer inspection rather than as direct evidence that the corresponding records are unreliable. This distinction underpinned our focus on discordant BINs, where the aim was to assess which species assignments were better supported by the available taxonomic and metadata information.

To curate discordant BINs, we opted for a heuristic approach rather than establishing a fixed rule‐based system, using the records' data and metadata together with the extracted features to assess species assignments, identify pseudo‐discordances, and flag potential inconsistencies. This approach allows the available record information to be considered on a case‐by‐case basis, but it is not infallible and necessarily involves a degree of subjective judgement. Manual assessment nevertheless remains important when resolving taxonomic ambiguities and discordances within molecular taxonomic units (Ferreira et al. 2024; Kühbandner et al. 2026; Lavrador et al. 2023; Magoga et al. 2022; Radulovici et al. 2021). Some degree of disagreement is also inherent to taxonomic practice, as different taxonomists may reach different conclusions in ambiguous cases (Thiele et al. 2021), and such differences may consequently be reflected in the taxonomic identifications recorded for individual reference barcodes. Different interpretations may also arise from the relative weight assigned to morphological and molecular evidence in species delimitation (Sharkey et al. 2021; Zamani et al. 2022). Together, these factors make the curation of discordant BINs a context‐dependent process that is difficult to fully standardize.

The continued growth of DNA barcode reference libraries creates a need for approaches that can help scale record inspection and curation (Ratnasingham et al. 2024; Rimet et al. 2021). Here, we evaluated a rule‐based approach, logistic regression, XGBoost, and ANN in their ability to approximate the manual curation of discordant BINs. This comparison allowed us to assess whether increasing model complexity improves the reproduction of manual curation decisions. The simpler approaches performed comparably well. The rule‐based method reached a maximum observed accuracy of 90.9% at the 80% majority threshold, while logistic regression achieved the highest numerical performance among the supervised classifiers (92.1%), followed closely by ANN (91.7%) and XGBoost (91.4%). The comparatively small differences among supervised machine learning approaches suggest that much of the information used during manual curation can be represented using relatively simple combinations of the available predictors, at least for the dataset considered here. The rule‐based results nevertheless also illustrate the limitations of relying on a small set of fixed criteria, as performance varied substantially depending on the majority threshold applied. Supervised approaches may therefore provide a more flexible way of combining several sources of record‐level and BIN‐level information without requiring a single predetermined decision rule. At the same time, the variation in performance across held‐out folds further supports a human‐in‐the‐loop approach, in which automated classifications assist rather than replace manual curation.

The grouped cross‐validation design used here was intended to provide a more stringent assessment than random record‐level partitioning. By assigning complete BINs to the outer test folds, records from the same BIN could not occur in both training and test data, and performance was therefore evaluated on BINs that were not used during model fitting or hyperparameter selection. This design is appropriate for assessing transfer to previously unseen BINs within the same dataset and taxonomic context, but it does not establish broader transferability. In particular, performance on new species or genera, other taxonomic groups, different barcode markers, or reference libraries with different structures may differ substantially from that observed here.

An important next question is whether incorporating additional biological information improves the classification of discordant BINs, and whether doing so changes the relative value of model flexibility. The predictors considered here consisted predominantly of structured variables derived from taxonomic assignments and associated metadata, and the manual classification procedure itself relied heavily on this type of information. Future curation frameworks could incorporate a broader range of predictors, including sequence‐derived features, measures of genetic divergence, phylogenetic or phylogeographic information, specimen‐level information, and potentially images. If such information improves classification, nonlinear relationships and interactions among predictors may become more relevant, potentially increasing the value of models such as XGBoost or ANNs. Deep‐learning approaches are already being explored for applications in molecular ecology, including species classification and biodiversity assessment (Lamperti et al. 2023; Müller et al. 2023; Yang et al. 2021), and may become increasingly relevant for barcode curation as richer biological information is incorporated.

The XGBoost permutation importance analysis further supports the interpretation that this model primarily reproduced patterns that are directly relevant to the manual curation procedure. The proportion of records within a BIN assigned to the focal species was by far the most influential predictor, while the number of unique identifiers, synonym status, and Shannon entropy showed smaller but still positive contributions. The remaining predictors had comparatively limited or inconsistent individual effects. This pattern is consistent with the importance placed during manual inspection on the relative representation of species names within a BIN, while also suggesting that the model drew additional predictive information from the number of unique taxonomic identifiers.

Overall, the high agreement between automated predictions and the manual labels indicates that these approaches can be useful for scaling the screening of discordant barcode records, particularly for cases with clearer supporting evidence. Their intended role is not to replace manual assessment or to establish the taxonomic correctness of individual records, but to provide a triage step that can help prioritize cases for closer inspection. Records classified as *supported* would therefore represent species assignments that are well supported by their representation within the BIN and the available record metadata, rather than definitive assessments of taxonomic correctness. Conversely, records classified as *inconclusive* can be prioritized for further examination. This type of decision‐support framework could reduce the amount of case‐by‐case inspection required as reference libraries continue to grow, while retaining human assessment for ambiguous or otherwise difficult cases.

Scaling such approaches to much larger and more taxonomically diverse reference libraries would nevertheless introduce additional challenges. These include the computational requirements associated with larger datasets, the increasing heterogeneity of taxonomic and metadata information, and, for supervised approaches, the effort required to obtain sufficiently representative manually curated records for model development. The accumulation of expert‐curated datasets across different taxonomic groups could help address this limitation and provide a stronger basis for evaluating the transferability of the approaches tested here. Tools such as the BGE Library Curation Tool (Kühbandner et al. 2026), which enables taxonomic experts to review individual barcode records, revise taxonomic information, and document curation decisions and their justification, will facilitate the generation of such structured curated datasets. As these annotations accumulate, they could provide useful training and evaluation data for future automated curation frameworks. More broadly, the challenges posed by increasingly large reference libraries are not limited to curation. Similar issues of scale also arise in taxonomic classification, where increasingly large reference libraries can substantially increase computational demands. PROTAX‐GPU illustrates one approach to addressing this bottleneck by using GPU‐based parallelization to accelerate sequence‐similarity and nearest‐neighbour calculations, enabling probabilistic DNA barcode identification against reference libraries containing millions of sequences (Li et al. 2024).

### 5.1 Representative Examples of Model Classifications

Representative cases illustrate how the different approaches handled distinct sources of BIN discordance. For example, BIN BOLD:AAB8987 contained the synonymous species names *Centroselachus crepidater* and *Centroscymnus crepidater* , both referring to the longnose velvet dogfish. In this case, the rule‐based approach produced the same classification by design. Similarly, all records were classified as *supported* by logistic regression, ANN and XGBoost. A more complex case was observed for BOLD:AAB6180, which included *Sardinops melanosticta* (Japanese pilchard) and *Sardinops sagax* (Pacific sardine), considered synonyms in WoRMS, together with a single potentially misidentified record of *Spratelloides robustus* (blue sprat). In this case, only XGBoost reproduced the complete manual classification, assigning the synonym records as *supported* while flagging the singleton as *inconclusive*. Cases involving ambiguous species names were also mostly handled correctly by the automated approaches. For example, BOLD:ACF1143 contained a majority of *Gadus morhua* (Atlantic cod) records, which were classified as *supported*, together with a single *Gadus* sp. record classified as *inconclusive* because it lacked a species‐level identification.

False negatives were also observed in some cases involving pseudo‐discordance due to synonymy (Figure 4). For example, BIN BOLD:AAE9134 comprised two synonymous species names referring to the So‐iuy mullet, *Planiliza haematocheila*, which accounted for 78.9% of the records in the BIN and had been identified by four different individuals, and *Planiliza haematocheilus*, for which information on the identifier was unavailable. XGBoost correctly classified the records associated with both names as *supported*, whereas ANN and logistic regression classified the minority synonym records as *inconclusive*. However, the ANN prediction was close to the classification threshold, assigning a probability of 48.6% to the *supported* class, compared with 36.4% for logistic regression. Synonymy was relatively uncommon in the manually curated dataset, occurring in only 40 of the 1138 discordant BINs, which may have limited the opportunity for the supervised models to learn how this pattern should be weighted relative to the other predictors. Importantly, although synonym status was included as a predictor, it was not imposed as a deterministic rule on the supervised classifiers. Such a rule could resolve known synonymies automatically, but its applicability ultimately depends on the completeness and currency of the underlying taxonomic resources. Databases such as WoRMS provide extensive information on accepted names and synonymies, but nomenclature is continually revised, and recently proposed or incompletely documented synonymies, spelling variants, or other taxonomic inconsistencies may not always be captured automatically. These cases may therefore still require human assessment.

All records with ambiguous species names were manually classified as *inconclusive* by design in the rule‐based approach. The supervised models generally reproduced this pattern, with one exception involving BIN BOLD:AAD4149. In this BIN, *Serrivomer* sp. (sawtooth eels) accounted for 89.5% of the records and was classified as *supported* by logistic regression and XGBoost, whereas ANN correctly classified these records as *inconclusive*. The three supervised methods also failed to reproduce the manual classification for BIN BOLD:AAB1178, which included 46 records of Sloane's viperfish, *Chauliodus sloani* , identified by 15 different individuals, alongside 85 additional records annotated as *Chauliodus* sp. followed by alphanumerical identifiers (e.g., *Chauliodus* sp. DPND_1895). While the *C. sloani* records were classified as *supported* during manual curation, logistic regression, ANN, and XGBoost classified them as *inconclusive*. In this BIN, the large number of records carrying distinct ambiguous names reduced the relative representation of *C. sloani* and created a pattern resembling the coexistence of multiple species assignments within the same BIN. Such cases illustrate how heterogeneous or inconsistent naming practices can complicate automated curation, even when the available record information supports a species assignment during manual inspection.

In other cases, however, multiple morphospecies names are indeed present within a single BIN. BOLD:AAA7352 provides such an example, including records from eight different morphospecies names of the *Thunnus* genus (tunas), a group for which interspecific genetic distances based on DNA barcodes can be very low (Lowenstein et al. 2009; Ward et al. 2005). In this and other similar scenarios, the automated approaches generally reproduced the manual classification, assigning the records as *inconclusive*. In these cases, the aggregation of morphospecies does not necessarily reflect operational inconsistencies or misidentifications but may instead result from the limited ability of COI variation and BIN clustering to discriminate among those morphospecies. When COI does not provide sufficient discrimination among closely related species, the available molecular evidence may only support identification at the genus level.

The three supervised models were able to reproduce the manual classification in examples involving putative incorrect species annotations. For example, BIN BOLD:AAA5956 consisted of 411 *Scomber scombrus* (Atlantic mackerel) records identified by 13 different individuals and two *Scomber japonicus* (chub mackerel) records for which information on the identifier was unavailable. The *S. scombrus* records were classified manually as *supported* and the *S. japonicus* records as *inconclusive*, a classification reproduced by logistic regression, ANN, and XGBoost. The rule‐based approach also reproduced this classification across all evaluated majority thresholds, given the strong predominance of *S. scombrus* within the BIN. Another example was BOLD:ADF5681, which included records from three snapper morphospecies: *Lutjanus fulviflamma* (dory snapper), *Lutjanus johnii* (John's snapper), and *Lutjanus monostigma* (one‐spot snapper). *L. fulviflamma* was identified by 17 individuals, was solely assigned to BOLD:ADF5681, and comprised 86.6% of the records in the BIN. In contrast, *L. johnii* was assigned to one additional BIN and had been identified by only one individual, while *L. monostigma* was assigned to two additional BINs and lacked information on the identifier. Logistic regression, ANN and XGBoost reproduced the manual classification by assigning *L. fulviflamma* as *supported* and the remaining records as *inconclusive*. In this case, however, the outcome of the rule‐based approach depended on the majority threshold: it reproduced the manual classification at thresholds up to 85%, whereas thresholds of 90% and 95% classified *L. fulviflamma* as *inconclusive*. These examples illustrate how strong agreement among several sources of record information can facilitate automated classification, while also showing how fixed rule‐based decisions may be sensitive to the threshold applied.

Although false positives represented a relatively small proportion of the overall classifications (Figure 4), some cases illustrate the difficulty of distinguishing a strong majority pattern from genuine taxonomic uncertainty. For example, BOLD:AAA1547 included two morphospecies, *Squalus acanthias* (spiny dogfish) and *Squalus suckleyi* (Pacific spiny dogfish), which are recognized as distinct but morphologically similar species (Ebert et al. 2010). *S. acanthias* accounted for 86.9% of the records in the BIN and had been identified by seven individuals across 13 institutions, whereas *S. suckleyi* accounted for 13.1% of the records but had nevertheless been identified by five individuals across eight institutions, suggesting that the minority assignment was also relatively well supported despite its lower representation. Despite the record predominance of *S. acanthias* , all records in this BIN were classified as *inconclusive* during manual curation because the available information suggested that discrimination between the two species remained uncertain. Logistic regression, ANN, and XGBoost instead classified the *S. acanthias* records as *supported* and the *S. suckleyi* records as *inconclusive*, thereby reproducing a pattern consistent with the relative abundance of the two species names within the BIN. The rule‐based approach showed a complementary limitation: thresholds from 55% to 85% produced the same majority‐driven classification as the supervised models, whereas the more stringent 90% and 95% thresholds classified both species as *inconclusive*, reproducing the manual classification. The example shows that the relative representation of species names within a BIN, although informative, does not fully capture more nuanced cases.

Importantly, false‐positive *supported* classifications are particularly relevant in a curation framework because such records may be less likely to receive further inspection than records classified as inconclusive. In practice, records classified as *supported* with lower confidence could also be retained for manual inspection. Future implementations could therefore adopt more conservative decision thresholds, probability‐based uncertainty zones, or explicit rules that direct borderline cases to manual review. However, the relatively high confidence associated with some false‐positive classifications indicates that such safeguards would not identify all problematic cases. These measures may nevertheless be valuable when the priority is to minimize the risk of retaining questionable assignments in reference libraries. Together, these examples illustrate both the potential of automated approaches for interpreting discordant barcode records and the challenges posed by particular cases.

## 6 Conclusion

DNA barcode reference libraries continue to expand rapidly (Hebert et al. 2025), creating an increasing challenge for maintaining taxonomic consistency at the scale required for both comprehensive and reliable species‐level identification. As the number of available records grows, so does the amount of information that must be inspected, reconciled, and interpreted. This challenge may be further complicated in cases where the morphological identification of reference specimens is itself uncertain. In this case study, a simple rule‐based approach and three supervised classifiers were able to reproduce a large proportion of the manual curation decisions made for discordant BIN records using taxonomic information and associated record metadata. Logistic regression achieved the highest numerical performance among the supervised approaches, while ANN and XGBoost performed similarly, indicating that increased model complexity did not provide a clear advantage for the predictors considered here. At the same time, the sensitivity of the rule‐based approach to the selected majority threshold and the behaviour of the supervised models in particular ambiguous cases illustrate why fixed rules or automated predictions alone may be insufficient in many cases of barcode discordance. These approaches are therefore better viewed as tools for triage and decision support, helping to scale the screening of increasingly large reference libraries while retaining human assessment for conflicting or uncertain cases.

Future work should test the transferability of this framework across broader taxonomic groups, independent datasets, and additional molecular markers, including 12S, which is widely used in fish metabarcoding (Xiong et al. 2022; Blanco et al. 2025). Where BINs are unavailable, analogous molecular units could be defined using alternative MOTU‐delimitation approaches based on genetic distance or phylogenetic relationships. More broadly, this study supports the development of reusable tools for triaging large molecular reference libraries. As barcode and metabarcoding datasets continue to grow, scalable curation will be essential to help ensure that gains in data volume are accompanied by improvements in the reliability of species‐level assignments.

## Author Contributions

J.T.F., P.S., F.O.C., and C.A.C. designed and conceived the research. J.T.F, A.O.F, and A.S.L. performed the manual data curation and labelling. J.T.F. wrote the programming code and performed data analysis with the help of C.A.C. J.T.F. wrote the manuscript with the help of all authors. All authors contributed to the manuscript and approved the final version.

## Funding

This work was funded by the projects ‘A‐FISH‐DNA‐SCAN: Cutting‐edge DNA‐based approaches for improved monitoring and management of fisheries resources along Magellan‐Elcano's Atlantic route’ (https://doi.org/10.54499/CIRCNA/BRB/0156/2019), funded by the Portuguese Foundation of Science and Technology (FCT, I.P. reference: CIRCNA/BRB/0156/2019), ‘FISH‐DNA‐MONITOR: Supporting monitoring and management of fisheries resources in Guinea‐Bissau through capacity‐building on cutting‐edge DNA‐based approaches’ (FCT AGA‐KHAN/541703280/2019) funded by the Portuguese Foundation of Science and Technology (FCT, I.P.) and Aga Khan Development Network and by the ‘Contrato‐Programa’ (https://doi.org/10.54499/UIDB/04050/2020), funded by national funds through the FCT I.P. J.T.F. (UI/BD/150910/2021; https://doi.org/10.54499/UI/BD/150910/2021) and A.S.L. (UI/BD/150871/2021; https://doi.org/10.54499/UI/BD/150871/2021) were supported by the Collaboration Protocol for Financing the Multiannual Research Grants Plan for Doctoral Students with financial support from FCT I.P. and the European Social Fund under the Northern Regional Operational Program—Norte2020. A.O.F. was supported by grant DFA/BD/6653/2020 (https://doi.org/10.54499/2020.06653.BD) from FCT I.P.

## Ethics Statement

This study used publicly available DNA barcode records and associated metadata and did not involve the collection or experimental manipulation of animals.

## Conflicts of Interest

The authors declare no conflicts of interest.

## Supporting information


**Data S1:** Grid‐search results, fold‐specific selected configurations, and training and validation curves for ANN and XGBoost.

**Data S2:** Record‐level predictions and additional model outputs, including fold‐specific results, test‐BIN assignments, fitted models and XGBoost permutation importance results.

**Table S1:** List of marine fish species used as queries to mine DNA barcode records from BOLD.

**Table S2:** Dataset of marine fish DNA barcode records and associated taxonomic information following qualitative grade assignment.

**Table S3:** Manually labelled dataset of records from discordant BINs used in the classification analyses.

## Data Availability

The programming scripts used in this study are publicly available on GitHub (https://github.com/tadeu95/ML‐Curation‐DNA‐Barcodes). Additionally, the data sets, models and associated files utilized in this study are available in the Supporting Information section of the manuscript.
